# Correlations of baseline neutrophil-lymphocyte ratio with prognosis of patients with lupus nephritis: A single-center experience

**DOI:** 10.2478/rir-2023-0029

**Published:** 2023-12-19

**Authors:** Yi Chen, Xue Wu, Xiaomei Chen, Mengmeng Li, Cainan Luo, Yamei Shi, Jing Li, Lijun Wu

**Affiliations:** Department of Rheumatology, People’s Hospital of Xinjiang Uygur Autonomous Region, Urumqi, Xinjiang Uygur Autonomous Region, China; Xinjiang Rheumatoid Arthritis Clinical Medical Research Center, Urumqi, Xinjiang Uygur Autonomous Region, China; Graduate School, Xinjiang Medical University, Urumqi, Xinjiang Uyghur Autonomous Region, China; Department of Rheumatology and Clinical Immunology, Peking Union Medical College Hospital, Chinese Academy of Medical Sciences & Peking Union Medical College, Key Laboratory of Rheumatology and Clinical Immunology, Ministry of Education, Ministry of Science & Technology, State Key Laboratory of Complex Severe and Rare Diseases, National Clinical Research Center for Dermatologic and Immunologic Diseases (NCRC-DID), Beijing, China

**Keywords:** neutrophil-lymphocyte ratio, lupus nephritis, renal function, prognosis

## Abstract

**Objective:**

We aimed to evaluate the correlations among the neutrophil-to-lymphocyte ratio (NLR), lupus nephritis (LN) clinical characteristics, and renal prognosis of patients with LN.

**Methods:**

We enrolled 122 patients who were diagnosed with LN at the Rheumatology Department of the People’s Hospital, Xinjiang Uygur Autonomous Region from January 2013 to April 2022. We determined the occurrence of renal adverse events in patients with LN by reviewing medical records and follow-up data. Correlations were analyzed using the Spearman test, and the quartile method was applied to classify all of the 122 patients who had completed follow-up into low, medium, and high NLR groups. The Kaplan–Meier survival curve was used to conduct survival analysis, and Cox regression analyses were used to explore possible potential risk factors.

**Results:**

The baseline NLR of patients with LN was positively correlated with C-reactive protein (CRP), serum creatinine, blood urea nitrogen, and systemic lupus erythematosus disease activity index scores (*P* < 0.05) and negatively correlated with estimated glomerular filtration rate (eGFR) and serum albumin (*P* < 0.05). Patients who completed follow-up were divided into three NLR groups based on their NLR values: 30 in the low (NLR ≤ 2.21), 62 in the medium (NLR > 2.21 and NLR ≤ 6.17), and 30 in the high NLR group (NLR > 6.17). The patient survival time before developing poor renal prognosis was significantly different among the three groups (*P* < 0.05). High NLR (hazard ratio [HR] = 3.453, 95% confidence interval [CI]: 1.260–9.464), CRP (HR = 1.009, 95% CI: 1.002–1.017), eGFR (HR = 0.979, 95% CI: 0.963–0.995), and 24-h proteinuria values (HR = 1.237, 95% CI: 1.025–1.491) as well as anti-double stranded DNA antibody positivity (HR = 3.056, 95% CI:1.069–8.736) were independent risk factors associated with a poor renal prognosis for patients with LN.

**Conclusion:**

The baseline NLR in peripheral blood can be used as a reference index for evaluating renal function and disease activity in patients with LN, and a high NLR has predictive value for the prognosis of patients with LN.

## Introduction

Systemic lupus erythematosus (SLE) is an autoimmune disease characterized by multi-organ damage. Lupus nephritis (LN) is the most common manifestation of kidney damage in SLE ^[1]^ up to 60% of adult patients with SLE suffer from LN.^[2]^ The pathogenesis of LN is considered complex and involves many factors, such as genetics, sex hormones, and the environment (*e. g*., viral and bacterial infection). Immune complex glomerulonephritis is caused by the deposition of immune complexes in glomerular capillary loops that activate complement.^[3,4]^ Although the understanding and treatment of LN are continually improving, up to 30% of patients with LN eventually progress to end-stage renal disease (ESRD),^[5]^ which is still the main cause of death in patients with SLE.^[6]^ Therefore, with regard to the individualized treatment of patients with LN, it is of great significance to accurately evaluate LN disease activity and predict renal function.

At present, the disease activity of SLE is evaluated using the SLE Disease Activity Index (SLEDAI); however, the method is complicated, and it is difficult to determine laboratory parameters. In comparison, routine blood examination alone is sufficient to calculate the neutrophil-to-lymphocyte ratio (NLR) easily and efficiently. Recent studies have shown that NLR can be used to evaluate the diagnosis, disease activity, therapeutic effect, and prognosis of tumors, cardiovascular diseases, and autoimmune disease.^[7, 8, 9]^ With regard to SLE research, it has been suggested that routine blood examinations can be used to predict organ damage in SLE, reflecting SLE disease activity, and for SLE prognosis.^[10,11]^ However, there are few reports on the application of NLR in LN, and its influence on renal function and prognosis in patients with LN has not been clarified. Therefore, this study aimed to evaluate the clinical significance of NLR in LN and provide reference data for clinical treatment.

## Patients and Methods

### Patients

This retrospective study included patients diagnosed with SLE in the Rheumatology Department of the People’s Hospital of Xinjiang Uygur Autonomous Region between January 1^st^, 2013 and April 30^th^, 2022 according to the 1997 American College of Rheumatology criteria.^[12]^ All patients were ≥18 years old, were diagnosed with LN confirmed by kidney biopsy, and the pathological types met the criteria based on the 2003 International Society of Nephrology/Renal Pathology Society classification system.^[13]^ Exclusion criteria included patients with incomplete medical records, those with other autoimmune diseases, malignant tumors, infectious diseases, or acute massive hemorrhage, and those who received blood transfusion treatment, hormone treatment, or immunosuppressant treatment within the last 4 months. Finally, 122 patients with completed follow-ups were included in the study, and the medical records of all patients were collected using the electronic medical record management system of the hospital.

This study was approved by the Ethics Committee of the People’s Hospital of the Xinjiang Uygur Autonomous Region (Approval Number: KY2021101507). This study conformed to the requirements of the Declaration of Helsinki. All patients provided verbal informed consent, and the requirement for written consent was waived.

### Data Collection

Demographic and clinical data of 122 patients were collected from their medical records in hospital information system. The laboratory data prior to any medical intervention at baseline was obtained at the day of the hospital admission for the first time. Laboratory data included white blood cell, neutrophil, lymphocyte, and platelet counts; hemoglobin level; erythrocyte sedimentation rate (ESR); C-reactive protein (CRP); complement 3; complement 4; 24-h proteinuria; serum albumin; serum creatinine; blood urea nitrogen (BUN); anti-double-stranded DNA (dsDNA) antibody; anti-Smith antibody; Additionally, use of drugs during treatment were obtained and baseline NLR; estimated glomerular filtration rate (eGFR); and SLEDAI scores. eGFR was calculated using the simplified Modification of Diet in Renal Disease formula: eGFR (mL/min/1.73 m^2^) = 186 × (serum creatinine)-^1.154^ × (age)-^0.203^ × (0.742 female).

### Patient Follow-up

Baseline data was defined as the day of first-time hospitalization. Patients were followed up either through reviewing the medical records or phone interviews to evaluate the renal prognosis of the patients. The follow-up endpoint time was May 1^st^, 2022, or when a poor renal prognosis event occurred. Poor renal prognosis was defined as progression to ESRD, the need for renal replacement therapy, or death. Survival time was estimated as the time from the baseline date to the follow-up end date.

### Statistical Methods

Statistical analysis was performed using SPSS26.0 (Shanghai Cabit Information Technology Co., Ltd., China) and Graphpad Prism8 (GraphPad Software Co., Ltd., San Diego, CA, USA) software. Continuous data with a normal distribution were presented as mean ± standard deviation, and those with a skewed distribution were presented as median (quartile) (M [P^25^, P^75^] ). The correlations between clinical parameters and the level NLR were analyzed using Spearman correlation analysis. Patients who completed follow-up were further divided into low, medium, and high NLR groups by the quartile method. The Analysis of Variance (ANOVA) test was used to compare normally distributed continuous data among these three groups, and the Bonferroni method was used for between group comparison. Kruskall-Wallis H test was used to compare data that was not normally distributed among three groups, and the Nemenyi method was used for between group comparison. The survival rate and median survival time for these three groups were calculated using the Kaplan-Meier method; then, survival curves were drawn. The log-rank test (univariate analysis) was used to compare the difference between groups. Factors with statistical significance in the univariate analysis were included in the Cox regression model for multivariate analysis. A *P*-value < 0.05 was considered statistically significant.

## Results

### Clinical Characteristics of Patients with LN

Among the 122 patients with LN, there were 16 men and 106 women, with a male-to-female ratio of 1: 6.63 and an average age of 36.93±12.14 years. The overall NLR of all patients was 3.38 (2.20, 6.17). Baseline Clinical characteristics of patients with LN are listed in Table 1.

**Table 1:** Clinical characteristics of LN patients

	***n* = 122**
Age, years	36.93±12.14
Sex, Male/Female	16/106
leukocyte, ×109/L	6.48 (5.42, 8.04)
Neutrophils, ×109/L	4.36 (3.50, 6.05)
Lymphocytes, ×109/L	1.37 ± 0.63
Platelet, ×109/L	216.24 ± 76.65
Hemoglobin, g/L	116.50 (96.75, 130.25)
NLR	3.38 (2.20, 6.17)
ESR, mm/h	33 (18, 58)
CRP, mg/L	3.48 (1.46, 10.48)
Serum creatinine, μmol/L	66.15 (51.68, 110.25)
BUN, μmol/L	6.20 (4.21, 11.47)
eGFR, ml/min	92.84 ± 51.28
Albumin, g/L	32.30 ± 10.89
Uric acid, μmol/L	321.06 ± 126.23
24-h proteinuria, g/24 h	1.75 (0.39, 3.20)
Complement 3, g/L	0.58 ± 0.28
Complement 4, g/L	0.12 (0.06, 0.20)
Positive Anti-dsDNA antibody, *n* (%)	46 (37.7)
Positive Anti-Smith antibody, *n* (%)	33 (27)
SLEDAI	8 (6, 12)
Medication	
Glucocorticoid, *n* (%)	122 (100)
Hydroxychloroquine, *n* (%)	108 (88.52)
Cyclophosphamide, *n* (%)	51 (41.80)
Mycophenolate mofetil, *n* (%)	54 (44.26)
Biological DMARDs, *n* (%)	16 (13.11)
Other DMARDs, *n* (%)	29 (23.77)

ESR, erythrocyte sedimentation Rate; CRP, C-reactive protein; BUN, blood urea nitrogen; NLR, neutrophil-to-lymphocyte ratio; eGFR, estimated glomerular filtration rate; ds-DNA, double stranded DNA; SLEDAI, Systemic Lupus Erythematosus Disease Activity Index

### Correlation Analysis between Clinical Parameters and NLR

**Figure 1:**
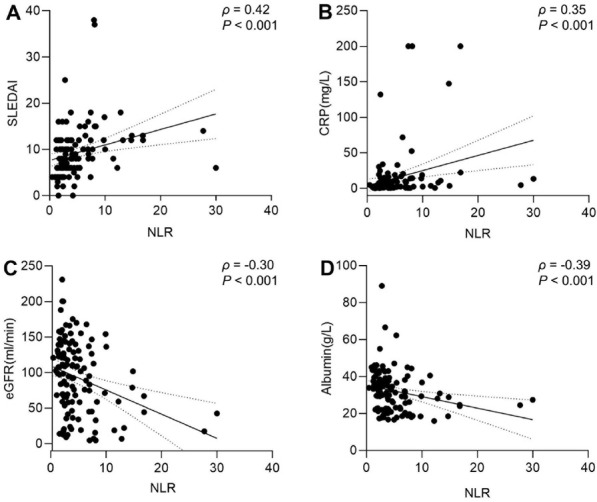
Correlation between NLR and SLEDAI score, CRP, eGFR, albumin in LN patient (Spearman correlation analysis). Note: A: NLR was positively correlated with SLEDAI score; B: NLR was positively correlated with CRP; C: NLR was negatively correlated with eGFR; D: NLR was negatively correlated with albumin (All P<0.05). NLR, neutrophil-to-lymphocyte ratio; SLEDAI, Systemic Lupus Erythematosus Disease Activity Index; CRP, C-reactive protein; eGFR, estimated glomerular filtration rate.

Spearman correlation analysis showed that baseline NLR was positively correlated with CRP (*ρ* = 0.35, *P* < 0.05), serum creatinine (*ρ* = 0.31, *P* < 0.05), BUN (*ρ* = 0.35, *P* < 0.05), and the SLEDAI score (*ρ* = 0.42, *P* < 0.05) and negatively correlated with eGFR (*ρ* = -0.30, *P* < 0.05) and serum albumin (*ρ* = -0.39, *P* < 0.05). There was no obvious correlation with other parameters (Table 2, Figure 1).

**Table 2:** Correlation analysis of LN patient clinical parameters and NLR

	**NLR**	
	***ρ***	***P*-value**
Hemoglobin, g/L	-0.27	0.003
ESR, mm/h	0.11	0.249
CRP, mg/L	0.35	<0.001
Serum creatinine, μmol/L	0.31	<0.001
BUN, μmol/L	0.35	<0.001
eGFR, ml/min	-0.30	0.001
Albumin, g/L (g/L)	-0.39	<0.001
Uric acid, μmol/L	0.20	0.031
24-hour proteinuria, g/24 h	0.19	0.033
Complement 3, g/L	-0.10	0.264
Complement 4, g/L	-0.07	0.464
Positive Anti-dsDNA antibody, *n* (%)	0.03	0.729
Positive Anti-Smith antibody, *n* (%)	0.07	0.474
SLEDAI	0.42	<0.001

ESR, erythrocyte sedimentation Rate; CRP, C-reactive protein; BUN, blood urea nitrogen; NLR, neutrophil-to-lymphocyte ratio; eGFR, estimated glomerular filtration rate; ds-DNA, double stranded DNA; SLEDAI, Systemic Lupus Erythematosus Disease Activity Index;

### Quartile Method of Group Comparison

Based on the NLR values, we classified 122 patients into three groups: 30 in the low (NLR ≤ 2.21), 62 in the medium (NLR > 2.21 and NLR ≤ 6.17), and 30 in the high NLR group (NLR > 6.17). Sex, hemoglobin level, NLR, CRP, serum creatinine, BUN, eGFR, serum albumin, SLEDAI score, and the poor renal prognosis rate were significantly different among the three NLR patient groups (*P* < 0.05) (Table 3).

**Table 3:** Clinical characteristics between different NLR groups

	**Low NLR group (*n* = 30)**	**Medium NLR (*n* = 62)**	**High NLR group (*n* = 30)**	**F/**χ**^2^/H**	***P*-value**
Age, years	33.60 ± 12.25	37.60 ± 11.75	38.87 ± 12.56	1.62	0.202
Sex, Male/Female	0/30	10/52*	6/24*	7.48	0.024
Hemoglobin, g/L	121.20 ± 14.44	117.5 (96, 131.5)	105.53 ± 28.89*	6.82	0.033
NLR	1.66 ± 0.42	3.55 ± 1.07*	8.12 (7.01, 12.90)*^Δ^	101.53	<0.001
ESR, mm/h	36.63 ± 23.63	37.45 ± 24.86	40.2 ± 27.60	0.17	0.842
CRP, mg/L	1.53 (1.15, 2.61)	4.24 (1.80, 10.12)*	9.30 (2.42, 20.85)*	14.58	0.001
Serum creatinine, μmol/L	54.95 (46.78, 64.85)	68.9 (54.18, 100.04)*	105.45 (69.55, 228.00)*^Δ^	15.46	<0.001
BUN, μmol/L	4.23 (3.61, 5.92)	6.33 (4.45, 10.83)*	11.55 (6.60, 16.35)*^Δ^	21.87	<0.001
eGFR, ml/min	118.97 ± 47.79	91.82 ± 48.60*	68.85 ± 49.15*^Δ^	8.03	0.001
Albumin, g/L	35.57 ± 7.16	31.73 (23.06, 37.42)*	27.40 ± 8.35	13.52	0.001
Uric acid, μmol/L	290 (240.75, 342.38)	321.95 ± 131.11	359.70 ± 128.65*	5.22	0.073
24-h proteinuria, g/24 h	1.15 (0.14, 2.55)	1.76 (0.54, 2.90)	2.80 ± 2.64	4.05	0.132
Complement 3, g/L	0.63 ± 0.32	0.59 ± 0.28	0.51 ± 0.22	1.40	0.250
Complement 4, g/L	0.15 (0.09, 0.21)	0.12 (0.06, 0.20)	0.12 ± 0.07	1.20	0.549
Positive Anti-dsDNA antibody, *n* (%)	13 (44.3)	22 (35.5)	11 (36.7)	2.97	0.637
Positive Anti-Smith antibody, *n* (%)	6 (20)	18 (29.0)	9 (30)	1.01	0.603
SLEDAI	6 (4, 10)	8 (6, 10)*	12 (9.75, 15)*^Δ^	25.62	<0.001
Poor renal prognosis, *n* (%)	1 (3.3)	8 (12.9)	12 (40)*^Δ^	15.80	<0.001

^*^P < 0.05 *vs*. the Low NLR group. ^Δ^*P* < 0.05 *vs*. the Medium NLR group. ESR, erythrocyte sedimentation Rate; CRP, C-reactive protein; BUN, blood urea nitrogen; NLR, neutrophil-to-lymphocyte ratio; eGFR, estimated glomerular filtration rate; ds-DNA, double stranded DNA; SLEDAI, Systemic Lupus Erythematosus Disease Activity Index; DMARDs, Disease Modifying Anti-Rheumatic Drugs;

### Survival Analysis

**Figure 2:**
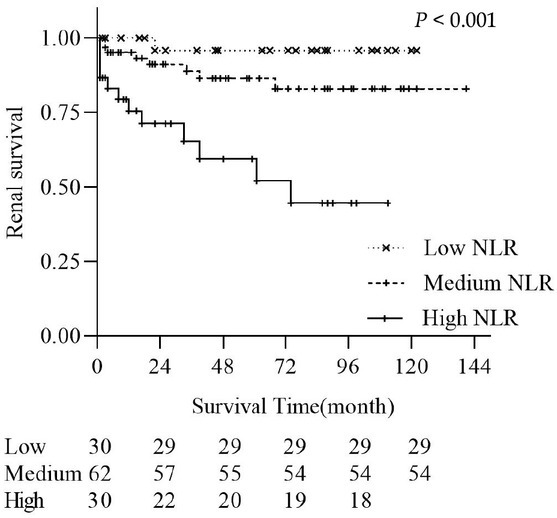
Kaplan–Meier curves of LN patients in different NLR groups. The overall rate of poor renal prognosis in three NLR groups was significant difference (P<0.05). NLR, neutrophilto-lymphocyte ratio.

According to our Kaplan–Meier analysis of the data, there was a significant difference in the overall rate of poor renal prognosis among the three NLR patient groups (*P* < 0.05) (Figure 2). In the low, medium and high NLR groups, the renal survival rates were 95.83%, 91.11%, and 71.28% at 24 months and 95.83%, 82.98%, and 59.40% at 48 months, respectively. There was a significant difference between the high NLR group and the medium and low NLR groups (*P* < 0.05); however, there was no significant difference between the medium and low NLR groups (*P* = 0.135).

### Regression Analysis

Univariate Cox regression analysis showed that sex, hemoglobin level, high NLR, ESR, CRP, eGFR, 24-h proteinuria, anti-dsDNA antibody positivity, and SLEDAI score were significantly associated with poor renal prognosis (*P* < 0.05), whereas multivariate Cox regression analysis showed that high NLR group (*vs*. Low and medium NLR group, hazard ratio [HR] = 3.453, 95% confidence interval [CI]: 1.260–9.464), CRP (HR = 1.009, 95% CI:1.002–1.017), eGFR (HR = 0.979, 95% CI: 0.963–0.995), 24-h proteinuria (HR = 1.237, 95% CI: 1.025–1.491) and anti-dsDNA antibody positivity (*vs*. negative anti-dsDNA antibody, HR = 3.056, 95% CI: 1.069–8.736) were independent risk factors associated with a poor renal prognosis in patients with LN (Table 4).

## Discussion

The pathogenesis of LN is unclear and may include autoantibodies, immune complex deposition, neutrophil extracellular traps (NETs), and innate and adaptive immune activation.^[14]^ Neutrophils play a key role in LN pathogenesis. They can infiltrate the kidneys and induce tissue damage through type I interferon secretion and NET production.^[15]^ Defective innate immune responses and abnormal activation of autoreactive T and B cells are important pathogeneses of SLE, and lymphocytopenia is a typical feature of SLE.^[16]^ NLR reflects neu-trophil and lymphocyte counts and as it is the combination of these two indicators, compared with each single indicator, it is less susceptible to physical, biochemical, or physiological factors and more valuable in predicting inflammation.^[17]^ Thus, NLR was chosen as the measure to be examined in the current study.

Through correlation analysis, we found that eGFR decreased with an increase in NLR, showing a negative correlation (*ρ* = -0.30), and serum creatinine and BUN increased with an increase in NLR, demonstrating a positive correlation (*ρ* = 0.31, *ρ* = 0.35). These results were consistent with the study by Xue *et al*.^[18]^ reporting that NLR was closely correlated with eGFR, and are also consistent with the results reported by Soliman *et al*.^[19]^ and Qin *et al*.^[20]^ regarding the relationship between NLR, BUN, and serum creatinine levels. Our present study further proved that the NLR is an important marker for assessing renal damage in patients with LN.

For patients with SLE, the assessment of disease activity is a key step in disease management. This study demonstrated that the SLEDAI score and CRP level increased with an increase in NLR, showing a positive correlation (*ρ* = 0.42, *ρ* = 0.35), whereas serum albumin level decreased with the increase of NLR, showing a negative correlation (*ρ* = -0.39). Studies have shown that CRP and ESR, non-specific inflammatory indicators, can be used to assess SLE disease activity or prognosis; it is possible for CRP and ESR to increase together and for one to increase when the other decreases.^[21]^ Serum albumin is the most abundant plasma protein and participates in the regulation of autoimmune diseases. Studies by Yip *et al*.^[22]^ and Idborg *et al*.^[23]^ showed that serum albumin levels are correlated with SLE disease activity. The present study found that the NLR value was closely correlated with CRP and serum albumin levels and could be used as an indicator of the inflammatory state. In recent years, studies have shown that NLR is a biomarker of various autoimmune diseases. Yang *et al*.^[9]^ conducted a comparative study of 1, 139 patients with autoimmune disease and 370 healthy controls and found that the NLR value increased in most connective tissue diseases (including SLE); therefore, NLR may be a useful tool that reflects the inflammatory state. Soliman *et al*.^[19]^ reported that the NLR value was positively correlated with the SLEDAI score, reflecting disease activity in SLE. These previous results were similar to the findings of our present study; however, the report by Liu *et al*.^[24]^ showed that there was no significant correlation between the NLR value and the SLEDAI score in patients with LN without complicated infections (*r* = 0.216). The reasons for these inconsistent results may be the geographical differences in the included samples and small sample size.

**Table 4:** Univariate and multivariate COX proportional hazard regression analysis of poor renal outcome in LN patients

	**Univariate**		**Multivariate**	
	**HR (95%CI)**	***P*-value**	**HR (95%CI)**	***P*-value**
Sex, Male *vs*. Female	0.346 (0.124-0.964)	0.042	0.418 (0.120-1.452)	0.170
Hemoglobin, g/L	0.979 (0.968-0.991)	<0.001	0.986 (0.965-1.007)	0.186
High NLR	5.512 (2.310-13.151)	<0.001	3.453 (1.260-9.464)	0.016
ESR, mm/h	1.021 (1.007-1.036)	0.004	0.992 (0.972-1.013)	0.447
CRP, mg/L	1.013 (1.007-1.020)	<0.001	1.009 (1.002-1.017)	0.010
eGFR, ml/min	0.975 (0.964-0.986)	<0.001	0.979 (0.963-0.995)	0.012
Albumin, g/L	0.966 (0.922-1.011)	0.138	-	
Uric acid, μmol/L	1.003 (0.999-1.006)	0.125	-	
24-h proteinuria, g/24h	1.249 (1.112-1.402)	<0.001	1.237 (1.025-1.491)	0.026
Complement 3, g/L	0.219 (0.044-1.092)	0.064	-	
Complement 4, g/L	1.109 (0.878-1.399)	0.386	-	
Positive Anti-dsDNA antibody	2.620 (1.102-6.229)	0.029	3.056 (1.069-8.736)	0.037
Positive Anti-Smith antibody	1.320 (0.533-3.271)	0.549	-	
SLEDAI	1.102 (1.048-1.158)	0.000	0.953 (0.866-1.048)	0.320

ESR, erythrocyte sedimentation Rate; CRP, C-reactive protein; NLR, neutrophil-to-lymphocyte ratio; eGFR, estimated glomerular filtration rate; ds-DNA, double stranded DNA; SLEDAI, Systemic Lupus Erythematosus Disease Activity Index; HR, hazard ratio; CI, confidence interval

ESRD is an important cause of death in patients with LN,^[25]^ and studies have shown that NLR can be used for ESRD risk assessment in patients with advanced CKD.^[26]^ Xue *et al*.^[18]^ followed-up 105 patients with LN for 6 months and found that NLR was a useful biomarker for predicting renal prognosis in these patients; however, few studies on LN have focused on the correlation between NLR and the prognosis of patient survival. In this study, patients were divided into low, medium, and high NLR groups. Baseline data showed differences in clinical indicators among the three groups, with patients in the high NLR group showing higher inflammatory markers and disease activity. Survival analysis showed a significant difference in the rate of poor renal prognosis among the three groups, with a higher rate in the high NLR group than in the medium and low NLR groups. Therefore, patients newly diagnosed with LN with a high NLR have a worse prognosis. Considering the sample size, we combined the medium and low NLR groups into one group in the Cox regression analysis for comparison with the high NLR group. A multivariate Cox regression model demonstrated that high NLR, CRP, eGFR, 24-h proteinuria, and anti-dsDNA antibody positivity were independent risk factors for poor renal prognosis in patients with LN, which is consistent with the findings of Xue *et al*.^[18]^ Therefore, NLR has clinical significance in recognizing survival prognosis in patients with LN, particularly in those who are newly diagnosed and having an NLR > 6.17. There is a high risk of disease progression; therefore, close treatment and follow-up are required.

This study has some potential limitations. This was a single-center retrospective study with a relatively small number of patients. Therefore, multicenter, prospective, and large-sample statistical evaluations and validations are required in future studies. Second, NLR may be easily affected by various medications and physicochemical factors, and we only excluded the influences of glucocorticoids and immunosup-pressants from the medical records, which inevitably have potential bias; Third, because of the incomplete collection of data, we did not evaluate the relationship between NLR and disease remission in this study. Fourth, we still need to continue follow-up to obtain long-term survival data to evaluate the relationship of NLR in LN prognosis.

In conclusion, this study confirmed that baseline NLR can be used as a reference index for evaluating renal function and disease activity in patients with LN and that a high NLR has predictive value for the prognosis that can be used for the better management of these patients.

## References

[1] Parikh SV, Almaani S, Brodsky S (2020). Update on Lupus Nephritis: Core Curriculum 2020. Am J Kidney Dis.

[2] Anders HJ, Saxena R, Zhao MH, Parodis I, Salmon JE, Mohan C (2020). Lupus nephritis. Nat Rev Dis Primers.

[3] Chinese Rheumatology Association (2020). National Clinical Research Center for Dermatologic and Immunologic Diseases; Chinese Systemic Lupus Erythematosus Treatment and Research Group. Zhonghua Nei Ke Za Zhi.

[4] Zhang H, Yang NS, Lu J (2021). [Recommendations for the diagnosis and management of lupus nephritis in China]. Zhonghua Nei Ke Za Zhi.

[5] Obrișcă B, Vornicu A, Procop A (2022). A Histology-Guided Approach to the Management of Patients with Lupus Nephritis: Are We There Yet?. Biomedicines.

[6] Lee YH, Choi SJ, Ji JD (2016). Overall and cause-specific mortality in systemic lupus erythematosus: an updated meta-analysis. Lupus.

[7] Kobayashi T, Ito K, Kojima T (2022). Pre-pembrolizumab neutrophil-to-lymphocyte ratio (NLR) predicts the efficacy of second-line pembrolizumab treatment in urothelial cancer regardless of the pre-chemo NLR. Cancer Immunol Immunother.

[8] Adamstein NH, MacFadyen JG, Rose LM (2021). The neutrophillymphocyte ratio and incident atherosclerotic events: analyses from five contemporary randomized trials. Eur Heart J.

[9] Yang Z, Zhang Z, Lin F (2017). Comparisons of neutrophil-, monocyte-, eosinophil-, and basophil- lymphocyte ratios among various systemic autoimmune rheumatic diseases. APMIS.

[10] Suszek D, Górak A, Majdan M (2020). Differential approach to peripheral blood cell ratios in patients with systemic lupus erythematosus and various manifestations. Rheumatol Int.

[11] Yu H, Jiang L, Yao L (2018). Predictive value of the neutrophil-tolymphocyte ratio and hemoglobin insystemic lupus erythematosus. Exp Ther Med.

[12] Hochberg MC (1997). Updating the American College of Rheumatology revised criteria for the classification of systemic lupus erythema-tosus. Arthritis Rheum.

[13] Weening JJ, D’Agati VD, Schwartz MM (2004). The classification of glomerulonephritis in systemic lupus erythematosus revisited. J Am Soc Nephrol.

[14] Yu C, Li P, Dang X (2022). Lupus nephritis: new progress in diagnosis and treatment. J Autoimmun.

[15] Whittall-García LP, Torres-Ruiz J, Zentella-Dehesa A (2019). Neutrophil extracellular traps are a source of extracellular HMGB1 in lupus nephritis: associations with clinical and histopathological features. Lupus.

[16] Sang A, Danhorn T, Peterson JN (2018). Innate and adaptive signals enhance differentiation and expansion of dual-antibody au-toreactive B cells in lupus. Nat Commun.

[17] Huang Z, Fu Z, Huang W (2020). Prognostic value of neutrophilto-lymphocyte ratio in sepsis: A meta-analysis. Am J Emerg Med.

[18] Lingyu Xue, Yanping Shi, Jing Zhang (2022). Correlations of peripheral blood neutrophil-lymphocyte ratio and lymphocytemonocyte ratio with renal function and prognosis in patients with lupus nephritis. Am J Transl Res.

[19] Soliman WM, Sherif NM, Ghanima IM (2020). Neutrophil to lymphocyte and platelet to lymphocyte ratios in systemic lupus erythematosus: Relation with disease activity and lupus nephritis. Reumatol Clin (Engl Ed).

[20] Qin B, Ma N, Tang Q (2016). Neutrophil to lymphocyte ratio (NLR) and platelet to lymphocyte ratio (PLR) were useful markers in assessment of inflammatory response and disease activity in SLE patients. Mod Rheumatol.

[21] Colombet I, Pouchot J, Kronz V (2010). Agreement between erythrocyte sedimentation rate and C-reactive protein in hospital practice. Am J Med.

[22] Yip J, Aghdassi E, Su J (2010). Serum albumin as a marker for disease activity in patients with systemic lupus erythematosus. J Rheumatol.

[23] Idborg H, Eketjäll S, Pettersson S (2018). TNF-α and plasma albumin as biomarkers of disease activity in systemic lupus erythematosus. Lupus Sci Med.

[24] Liu P, Li P, Peng Z (2020). Predictive value of the neutrophil-tolymphocyte ratio, monocyte-to-lymphocyte ratio, platelet-to-neutrophil ratio, and neutrophil-to-monocyte ratio in lupus nephritis. Lupus.

[25] China Lupus Nephritis Diagnosis And Treatment Guidelines Writing Group (2019). Chinese guidelines for the diagnosis and treatment of lupus nephritis. National Medical Journal of China.

[26] Yuan Q, Wang J, Peng Z (2019). Neutrophil-to-lymphocyte ratio and incident end-stage renal disease in Chinese patients with chronic kidney disease: results from the Chinese Cohort Study of Chronic Kidney Disease (C-STRIDE). J Transl Med.

